# LRSim: A Linked-Reads Simulator Generating Insights for Better Genome Partitioning

**DOI:** 10.1016/j.csbj.2017.10.002

**Published:** 2017-11-09

**Authors:** Ruibang Luo, Fritz J. Sedlazeck, Charlotte A. Darby, Stephen M. Kelly, Michael C. Schatz

**Affiliations:** a Department of Computer Science, Johns Hopkins University, United States; b Center for Computational Biology, McKusick-Nathans Institute of Genetic Medicine, Johns Hopkins University School of Medicine, United States; c Center for Health Informatics and Bioinformatics, New York University School of Medicine, United States; d Simons Center for Quantitative Biology, Cold Spring Harbor Laboratory, United States

**Keywords:** Linked-read, Molecular barcoding, Reads partitioning, Phasing, Reads simulation, Genome assembly, 10X Genomics

## Abstract

Linked-read sequencing, using highly-multiplexed genome partitioning and barcoding, can span hundreds of kilobases to improve *de novo* assembly, haplotype phasing, and other applications. Based on our analysis of 14 datasets, we introduce LRSim that simulates linked-reads by emulating the library preparation and sequencing process with fine control over variants, linked-read characteristics, and the short-read profile. We conclude from the phasing and assembly of multiple datasets, recommendations on coverage, fragment length, and partitioning when sequencing genomes of different sizes and complexities. These optimizations improve results by orders of magnitude, and enable the development of novel methods. LRSim is available at https://github.com/aquaskyline/LRSIM.

## 1 Background

Haplotype-resolved or phased genomes are desirable for obtaining insight into diploid or polyploid genomes and studying allele specific expression, allele-specific regulation, and many other important genomic features [1]. However, most of the genomes assembled to date are only a single haploid ‘mosaic’ consensus sequence with parental alleles merged arbitrarily [2]. Only a few studies have reported true diploid *de novo* assemblies so far. One of the first such studies successfully combined Illumina short-read sequencing, PacBio sequencing, and BioNano Genomics, generated a phased assembly of the widely studied human sample NA12878 [3]. The second introduced the FALCON-Unzip algorithm and applied it to *de novo* assemble three phased diploid genomes of *Arabidopsis thaliana*, *Vitis vinifera* (grape), and the coral fungus *Clavicorona pyxidata*, relying exclusively on PacBio sequencing [4]. A third approach generated 605,566 fosmid clones on the YH1 human genome and mixed them into 30 clones per pool, each pool containing 0.04% of the diploid genome [5]. However, the sample requirements and the associated high cost of the three studies preclude their widespread use.

More recently, Zheng et al. demonstrated a high-throughput low-cost method marketed as GemCode and its successor Chromium by 10X Genomics for creating human reference-based phased genomes [6]. It uses an automated microfluidic system to isolate high molecular weight DNA molecules inside millions of partitions containing sequencing primers and a unique barcode to prepare a library that can be used for Illumina paired-end read sequencing. Each partition contains several DNA molecules spanning up to 100 kbp or more, and will share the same barcode. The library can be generated with as little as 1 ng of high molecular weight DNA, far less than alternative approaches. And as the sequencing is performed using inexpensive Illumina short-read sequencing, the overall cost is at least two orders of magnitude lower than pooled fosmid sequencing for a diploid genome and significantly less expensive than current long-read sequencing alternatives [7]. The method has been widely used to study novel species such as the Hawaiian Monk Seal [8] and the Pepper [9], as well as additional resequencing analysis in human samples [10].

Simulated sequencing data has proved indispensable for guiding tool development and evaluating tool performance [11]. Especially for the complex and unique workflow involved in constructing linked-reads, it is essential to develop simulation software that can produce linked-reads that capture the most essential characteristics of genome partitioning. To our knowledge, no alternative read simulator for linked-reads is available. Thus, we developed LRSim, a Linked-Reads Simulator, which simulates whole genome sequencing modeled on Chromium Linked-Read technology but is flexible enough to represent alternative technologies. We modeled the characteristics of all the relevant steps of the Chromium protocol so that it can be used to study linked-read sequencing of different genomes, mutation rates, input libraries, and short-read sequencing conditions *in silico*. We tested LRSim with the 10X Genomics LongRanger variant identification and phasing application and the 10X Genomics SuperNova genome assembler [7] as well as the independent HapCUT2 phasing algorithm [12] to confirm that alignment, variant identification, phasing, and *de novo* assembly are supported and deliver results similar to those of real data. After studying simulated datasets with multiple parameter combinations, we concluded that 1) the best phase block size of human genome with 50x linked-reads sequencing coverage and 1.5M partitions (barcodes) can be achieved with a molecule size between 150 kbp and 200 kbp; and 2) the standard library preparation protocol tailor-made for the mammalian-sized genomes needs to be adjusted regarding the number of partitions (barcodes) before it can be efficiently used for other genomes of significantly different size, such as *A*. *thaliana*. We also recognize that linked reads can produce less contiguous genome assemblies than long reads in highly repetitive genomes like maize.

## 2 Results

### 2.1 Characteristics of Real 10 × Genomic Sequences

**Fig. 1:**
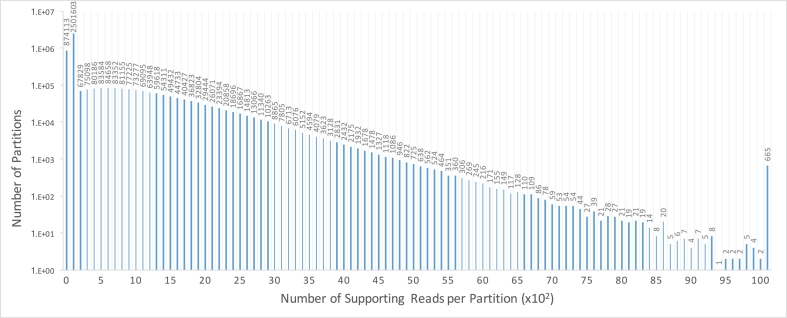
The distribution of number of supporting reads per partition. About 1.5 million partitions are supported by more than 100 reads. The rightmost column shows the number of partitions with the number of supporting reads per partition > 100.

We analyzed 13 publicly available real datasets processed by Chromium's LongRanger analysis pipeline to derive models and characteristics (Additional file 1: Supplementary Note). We identified ~ 1.5 (1.42) million partitions with > 100 reads each in NA12878 (Fig. 1). The Chromium uses 16 bp barcode sequence. The barcodes are located at the beginning of reads, thus having a higher error rate compared with the non-barcode bases. We observed 2.09% total base errors, which is slightly higher than the 1.78% estimated by base quality (Additional file 1: Supplementary Table 1). Interestingly, two of the barcodes were found to be consistently over-represented in all 13 samples (“GTATCTTCAGATCTGT”, “GTGCCTTCAGATCTGT”, Additional file 1: Supplementary Table 2).

**Fig. 2:**
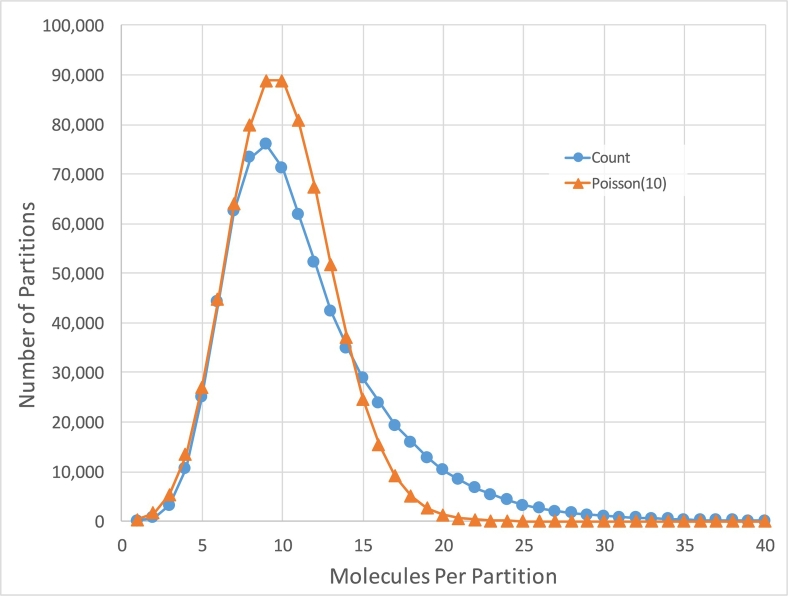
Distribution of the number of molecules per partition for NA12878.

**Fig. 3:**
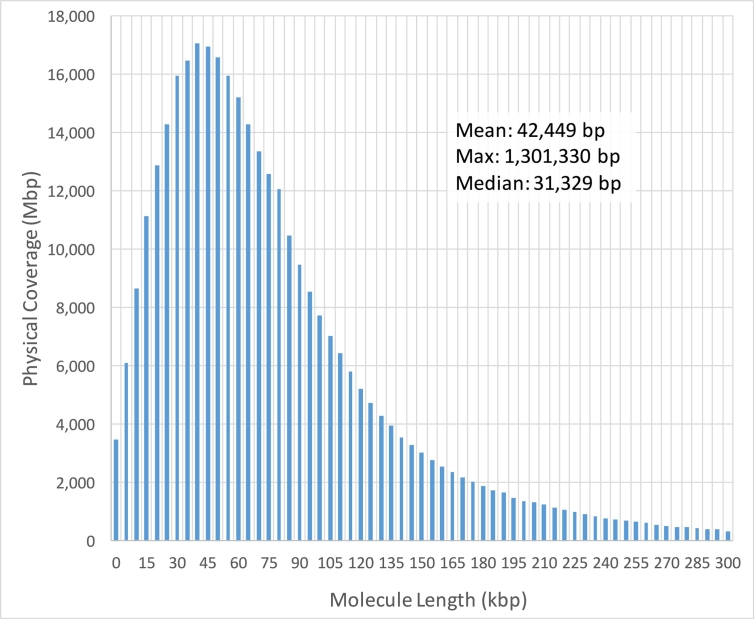
Weighted molecule length distribution for NA12878. Physical Coverage equals $\sum_{i=1}^{n}l_{i}$, where *l*_*i*_ is the length of a molecule and *n* is the number of molecules in that size range.

**Fig. 4:**
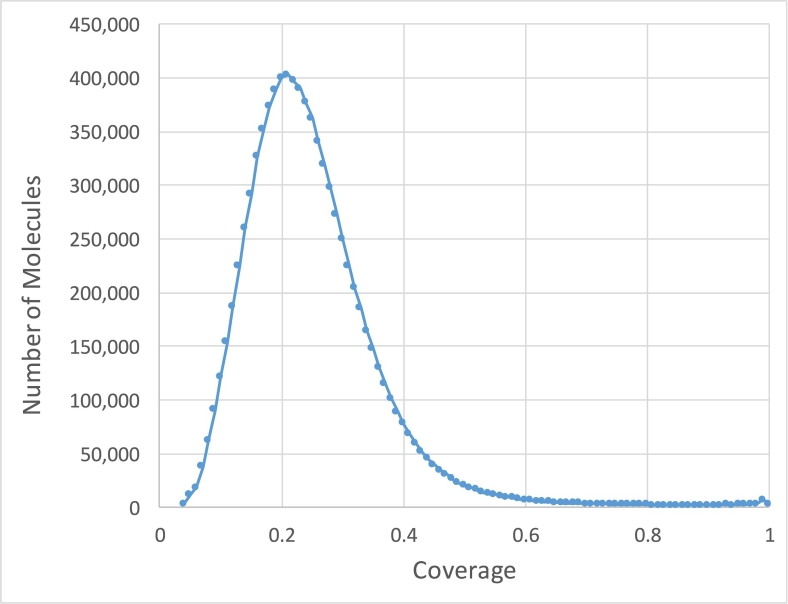
Distribution of molecule coverage for NA12878.

**Fig. 5:**
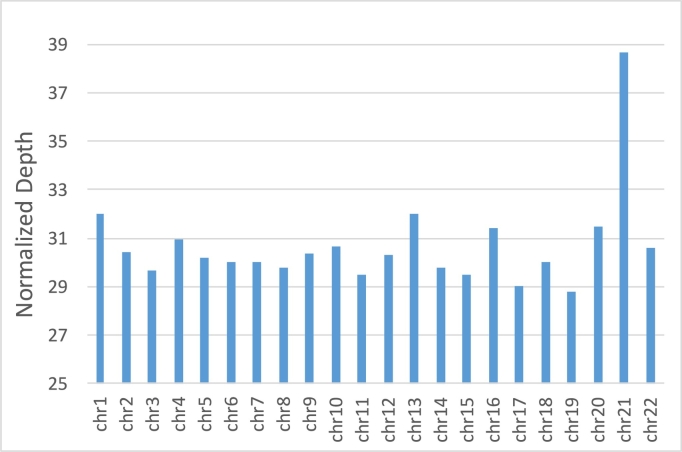
Average sequencing coverage of 13 samples per chromosome. The coverages were normalized to the sample with the lowest average coverage (NA24149, 30.36x).

One of the advantages of linked-reads is that they can be mapped into repetitive sequences not accessible by standard short read sequencing. Considering all regions ≥ 50 base-pairs that had zero coverage (excluding ambiguous genomics regions denoted by ‘N’), we found that the Chromium 80x NA12878 dataset left 164 Mb (5.47%) uncovered by any read alignment, versus 209 Mb (6.97%) that had zero coverage in the NIST 300x NA12878 Illumina only paired-end read dataset (Additional file 1: Supplementary Table 3, considering all alignments including MQ0). In the NA12878 sample specifically, a median 10 DNA molecules were allocated to each partition (Fig. 2) and the weighted molecule length peaked at around 40-50 kbp (Fig. 3). The distribution of molecule coverage peaked at 0.2x coverage (Fig. 4). Reads were generally uniformly distributed along the genome, as well as along the molecule, although surprisingly, we observed the chromosome 21 with unexpectedly high coverage in all samples possibly due to the existence of abundant Ribosomal DNA repeats around 9Mbp in the chromosome 21 according to the Gencode v26 gene annotations [13] and LongRanger analysis pipeline's way to deal with repetitive segments (Fig. 5; detailed views in Additional file 1: Supplementary Fig. 1a, b).

### 2.2 Parameters of Linked-Read

The overview of the technology for generating linked-reads and the available parameters of linked-reads were introduced in Zheng et al. [6]. By checking the variability of the available parameters in the 13 real datasets, we identified four important parameters and a dependent parameter of linked-read. The important parameters are: number of read pairs (*x*); number of partitions (*t*); mean molecule length (*f*); and mean number of molecules per partition (*m*). The dependent parameter is sequencing coverage per molecule (*c*). Given a genome size and a fixed *x*, the other four parameters *t*, *f*, *m* and *c* correlate with each other inversely. For example, with *x*, *f* and *m* remain constants, a higher number of partitions (*t*) will lead to lower sequencing coverage per molecule (*c*). The relationships between the parameters are introduced starting from the next subsection.

### 2.3 Effect of Molecule Size (f)

**Fig. 6:**
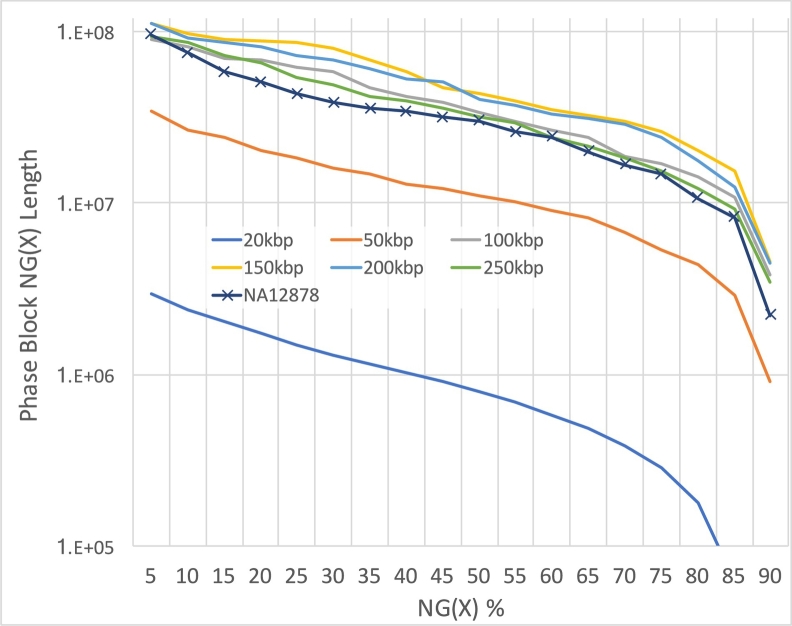
NG graph showing an overview of phased block sizes of 7 datasets. NG(X) is defined as X% of the genome is in phased blocks equals to or larger than the NG(X) length.

One of the critical requirements of linked-read construction is extracting high-quality, high-molecular weight DNA from the sample. To study how the molecule size changes the performance of linked-read sequencing, using human reference genome GRCh38, we simulated six datasets of different mean molecule sizes (*f*: 20, 50, 100, 150, 200 and 250 kbp), with 600 million read pairs (*x*), 1.5 million partitions (*t*) and 10 molecules per partition (*m*). Here we are using a normal distribution for the molecule lengths, although LRSim allows for arbitrarily complicated distributions as well. Instead of simulating random variants using SURVIVOR [14], which may not mimic the characteristics of real variants, we used 3.2M phased SNPs and indels identified from NA12878 (Additional file 1: Supplementary Note). The datasets were processed by LongRanger and phased by HapCUT2 [12] using a 48-core Intel E7–8857 v2 @3GHz machine with 1TB memory, running on average 1.5 days each. The sum of bases of different phase block sizes for six simulated datasets and NA12878 (Additional file 1: Supplementary Note) are shown in Fig. 6. The results show that the NA12878's performance lies between molecule size 50 kbp and 100 kbp, corroborating our observation in the weighted molecule length distribution of NA12878 (Fig. 4). The divergence between 50 kbp and NA12878 can be explained by the fact that the real data is platykurtic and has a longer tail on long molecules, which is an outcome that highly depends on the quality and length of DNA input.

We further noted that the Phase Block N50 sizes did not monotonically increase with longer molecules and instead plateaued at 200 kbp molecules. On investigation, we determined the cause to be that given a constant number of total reads, the coverage per molecule decreases proportionally to the molecule size. For example, if we increase the molecule size from 50 kbp to 250 kbp, the coverage decreases from ~ 0.2x to ~ 0.04x, and the average distance and standard deviation of the distances between reads rises, thus leading to shorter phase blocks.

### 2.4 Effect of the Number of Partitions (t)

We simulated six datasets with varying numbers of partitions (*t*), including 15k, 20k, 30k, 50k, 100k and the standard 1.5 million, to study the impact of the number of partitions on the assembly using the *A*. *thaliana* genome (TAIR10).

We kept the other parameters constant at 18 million read pairs (*x*), 50 kbp mean molecule size (*f*) and 10 molecules per partition (*m*). Using the same computer, SuperNova finished each assembly within 2 hours. The best assembly, as measured by contig N50, scaffold N50 or Phase Block N50, was from the dataset with *t* = 20,000 partitions (Table 1). Intriguingly, using 1.5 million partitions, which is the default for the 10X Chromium platform, the results are orders of magnitude worse (e.g. 14.6 kb vs. ~ 2 Mbp scaffold N50) for this reduced genome size.

**Table 1:** Assembly statistics of different number of partitions.

No. of partitions (× 1,000)	Contig N50	Phase Block N50	Scaffold N50
15	198,485	1,146,590	1,016,017
20	265,543	2,881,040	2,796,090
30	230,711	1,945,051	1,880,870
50	215,743	1,472,710	1,459,816
100	177,635	1,471,806	1,271,685
1500	14,597	1588	14,685

The Contig N50, Phase Block N50 and Scaffold N50 of the *A*. *thaliana* genome with 6 different partition numbers.

One of the major reasons for the deficiency in the assembly performance is attributed to insufficient coverage per molecule. For a 3 Gbp genome with the default parameters, the coverage per molecule is 0.2x on average. For *A*. *thaliana*, where the genome size is 20 times smaller (150 Mbp vs. 3 Gbp), if 3 parameters including *f*, *t* and *m* remain the default, the number of reads allotted to each molecule will be 20 times smaller, *i*.*e*. 0.01x coverage. The excessively low coverage increases the mean distance and its standard deviation between reads, which confounds the genome assembler; it also largely removes the chance for reads belonging to the same molecule to cover multiple heterozygous variants, which is essential for phasing. Therefore, we suggest adjusting the number of partitions according to the genome size if possible. On the current 10X Chromium instrument, it is not possible for the operator to directly control the number of partitions used, so alternatively we and 10 × Genomics recommend increasing the overall sequencing coverage and subsample the partitions proportionally. This approach also results in sufficient coverage per partition, which greatly improves the assembly results using linked-reads for smaller genomes.

### 2.5 Effect of Sequencing Coverage (x)

Genome assembly using Illumina short reads requires careful control of the sequencing coverage. Shallow coverage decreases the maximum usable kmer-size (to achieve the minimum requirement for kmer depth), thus limiting the ability to disentangle repetitive sequences. Excessively deep coverage leads to a lower signal-to-noise ratio because of the saturation of authentic sequences and the accumulation of more random errors in the ‘assembly graph’, thus decreasing the performance of the assembly outcome [15], [16]. The best practice for sequencing coverage ranges from around 30x to 100x coverage, depending on affordability (a longer kmer-size can be used with greater sequencing coverage, but this is limited by the length of read input and read errors) and the genomic nature of different species, including their heterozygosity, heterogeneity and repetitiveness. It is less clear how the sequencing coverage of linked-reads changes the performance of genome assembly.

Using the *A*. *thaliana* genome, we simulated four datasets with three different numbers of read pairs (*x*), 9, 18 and 27 million, which equates to 17-, 34- and 51-fold of the genome, respectively, and two different numbers of partitions, 20,000 and 30,000 for *x* = 27. The molecule length (*f*) was held constant at 50 kbp, as was the number of molecules per partition at *m* = 10. We used SuperNova to assemble the four datasets. The results are shown in Table 2. We found that 18 million read pairs (34-fold) with 20,000 partitions achieved the best Contig N50, Phase Block N50 and Scaffold N50. Interestingly, the assembly result of 27 million read pairs (51-fold) was worse than 18 million on all three metrics, and only improved slightly on Contig N50 and Phase Block N50 after increasing the number of partitions to 30,000 (to keep the molecule coverage the same as 18 million read pairs). This indicates that the sequencing coverage itself rather than the molecule coverage makes a difference in linked-reads genome assembly using the SuperNova assembler.

**Table 2:** Assembly statistics of different sequencing coverage.

No. of read pairs (M)	No. of partitions (× 1,000)	Contig N50	Phase Block N50	Scaffold N50
9 (17-fold)	20	233,233	1,027,768	899,826
18 (34-fold)	20	265,543	2,881,040	2,796,090
27 (51-fold)	20	221,680	1,971,701	1,896,517
27 (51-fold)	30	241,319	1,979,723	1,688,453

Contig N50, Phase Block N50 and Scaffold N50 of the *A*. *thaliana* genome with 4 different combinations of number of read pairs and number of partitions.

### 2.6 Effect of the Number of Molecules per Partition (m)

The number of molecules per partition is usually determined by sample preparation technologies and cannot be easily modified except by carefully controlling the total amount of input DNA. Thus, the number needs to be carefully selected and verified before production. A lower number of molecules per partition requires a larger number of barcodes to arrive at the same number of molecules. A higher number of molecules per partition requires fewer barcodes, but increases the chance of two molecules coming from the two haplotypes in the same genome position forming a “collision” that can lead to phase errors or reduced phase block sizes. Given a certain number of barcodes, the number of molecules per partition will increase or decrease the coverage per molecule, which changes the performance of genome assembly and phasing.

Using the *A*. *thaliana* genome, we simulated 6 datasets with 1, 4, 7, 10, 15 and 20 molecules per partition, with the number of read pairs (*x* = 18 million), molecule length (*f* *=* 50,000) and number of partitions (*t* = 20,000). The assembly results are shown in Table 3. The Phase Block N50 and Scaffold N50 peaked with 10 molecules per partition, while Contig N50 peaked with 7. The metrics change insignificantly in the range 4 to 20 molecules per partition and went down significantly with only 1 molecule per partition; the reason remains unclear. We speculate that it is related to the average molecule coverage increasing to 2x with only 1 molecule per partition; this could confound phasing algorithms oblivious to conflicting alleles caused by sequencing error within a molecule. Also, the total span of the genomic regions being covered decreases with the same number of partitions but less molecules per partition.

**Table 3:** Assembly statistics of different number of molecules per partition.

No. of molecules per partition	Contig N50	Phase Block N50	Scaffold N50
1	46,993	72,400	54,769
4	249,371	2,105,517	2,020,687
7	274,133	1,869,856	2,074,127
10	265,543	2,881,040	2,796,090
15	232,708	2,860,223	2,416,675
20	245,894	1,920,175	1,878,113

Contig N50, Phase Block N50 and Scaffold N50 of the *A*. *thaliana* genome with 6 different molecule numbers per partition.

### 2.7 Validation of LRSim in a Repetitive Plant Genome

**Fig. 7:**
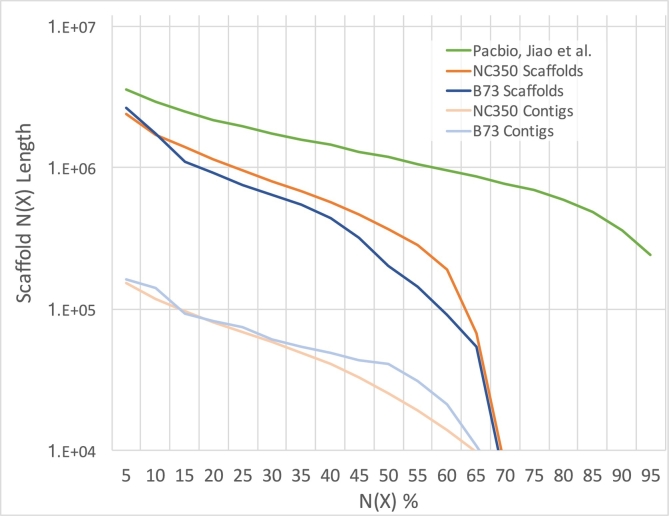
NG graph showing an overview of scaffold sizes of three datasets, including a simulated linked-reads for B73, real linked-reads for NC350 and a B73 assembly using PacBio long reads by Jiao et al. NG(X) is defined as X% of the genome is in phased blocks equals to or larger than the NG(X) length.

To illustrate the versatility of LRSim, we also analyzed linked-read sequencing of the highly repetitive maize genome line NC350 (SRA accession number PRJNA380806). NC350 is a temperate-adapted all-tropical inbred with broad adaptation and has a genome size of about 2.3 Gbp. Assembling 1036.45 million reads from this sample with SuperNova results in an assembly with a 25.68 kbp contig N50, 0.23 Mbp Phase Block N50 and 0.35 Mbp scaffold N50 (Fig. 7). Assembling the same number of simulated reads from the high-quality reference, established from a related line B73, results in a 41.03 kb Contig N50, 0.21 Mbp Phase Block N50 and 0.16 Mbp scaffold N50. While B73 is the closest species with an available reference genome, the modestly larger Contig N50 and modestly smaller scaffold N50 of the simulated data reflect the differences between the two genomes (NC350 vs. B73). Simulating reads from the incomplete reference genome also introduces some bases since linked reads will never span beyond the available contigs even though they would in the real sample. Nevertheless, a detailed comparison in Fig. 7 on both the contig and scaffold length distributions shows that the simulated data perform on par with the real data across the entire size range. In the figure, we also illustrate a Pacbio long read based assembly of the B73 genome [17] that demonstrates substantially higher contiguity than the linked-reads based B73 and NC350 scaffolds. These results indicate that a deficiency in assembly performance is expected when using the 10x Genomics' Chromium technology or other similar linked-reads technologies on genomes as repetitive and complicated as Maize.

## 3 Discussion and Conclusions

In this paper, we presented an analysis of 14 real datasets of linked-reads generated by 10x Genomics technology. Based on this analysis, we implemented a linked-read simulator named LRSim to allow fine tuning of both the type and number of variants and Illumina read specifications, and full control of important parameters for linked-reads we identified in real datasets, including 1) the number of read pairs; 2) the number of partitions; 3) the mean molecule length; and 4) the mean number of molecules per partition. We validated the performance of the simulator by the high concordance to the phasing and assembly results in the real NA12878 human dataset as well as in the highly repetitive maize genome. We concluded that from the phasing results of 6 simulated datasets with different mean molecule lengths and a real dataset of NA12878 that if constrained at a certain sequencing coverage, the best molecule size to achieve the best phase block size needs to be meticulously chosen. This can be done by wet-lab experiments, but would be more efficient with a simulator *in silico*. We also performed experiments on 6 simulated *A*. *thaliana* datasets with a different number of partitions and demonstrated a substantial degradation in assembly performance with an improper number of partitions, which leads to insufficient coverage per molecule. We concluded an appropriate sequencing coverage needs to be chosen for different applications and species before sequencing to achieve the best performance out of linked-reads.

In our study, linked-reads enabled much longer contigs, scaffolds and phase blocks on both the human genome and *A*. *thaliana* than using Illumina short-reads for genome assembly. The better outcomes, in turn, broaden the horizons for studies of allele specific expression, allele-specific regulation, and many other important genomic features critical to precision medicine. Furthermore, numerous other sequencing applications, such as improved structural variation analysis, epigenetics, metagenomics and RNA-seq, could potentially benefit from linked-read data. Linked-read technology is promising, and we believe that more complex genomics workflows will include and benefit from it. In 10x Genomics technology, “the number of partitions” of a certain product (such as GemCode and Chromium) is predetermined. Users can change two variables including “the molecule length” and “the number of read pairs”, and a dependent variable “the number of molecules per partition” on the total amount of DNA loaded for the experiment. We therefore encourage users to use LRSim to aid in the development of these new workflows and fully utilize the potential of the new linked-read technologies such as 10X Genomics and IGenomX [18], which follow similar molecular preparations but having very different properties with respect to the number of possible partitions and barcodes possible.

## 4 Methods

### 4.1 Identify Mismatches in Barcodes, the Number of Partitions, Molecule Lengths, Number of Molecules per Partition and Molecule Coverages in 13 Real Datasets

The BAM (Binary sequence Alignment/Map format) file of 13 samples generated by LongRanger was downloaded from the links in Additional file 1: Supplementary Note. For each pair of a paired-end read in a BAM file, the RX tag gives the raw barcode sequence, which is subject to sequencing errors. The BX tag gives the barcode sequence that is error-corrected and confirmed against a list of known-good barcode sequences. Differences between the two barcode sequences provided by RX and BX tag respectively are identified as mismatches in barcodes. The position, allele and base quality are extracted from the mismatches to study the error profile of barcodes.

Using the barcode sequences provided by the BX tags, reads were grouped according to their corresponding barcode sequence. Each group of reads were assembled using the ‘targetcut’ command of SAMtools [19]. Notice that the default parameters of ‘targetcut’ were optimized for Fosmid Pooling. Since a typical molecule coverage of linked-read sequencing is below 1, which is much lower than Fosmid Pooling, the parameters need to be fine-tuned to allow gaps in the assembled molecules. The detailed commands and a set of parameters empirically optimized for linked-read sequencing are presented in Additional file 1: Supplementary Note. The total number of partitions is determined as the number of unique barcodes in BX tags. Molecule lengths are the length of assembled sequences. The number of molecules per partition are the number of assembled sequences per partition. The molecule coverages are calculated as the percentage of non-gap regions of the assembled sequences.

### 4.2 Simulator Design and Performance

We included the following parameters in our simulator: *x*: number of read pairs; *t*: number of partitions; *f*: mean molecule length; and *m*: mean number of molecules per partition. The Poisson distribution is used to sample data from *f* and *m* by default; it can easily be switched to other functions. LRSim also allows for fine control of the short-read sequencing error profile and for biological variants to be introduced into the reference genome.

**Fig. 8:**
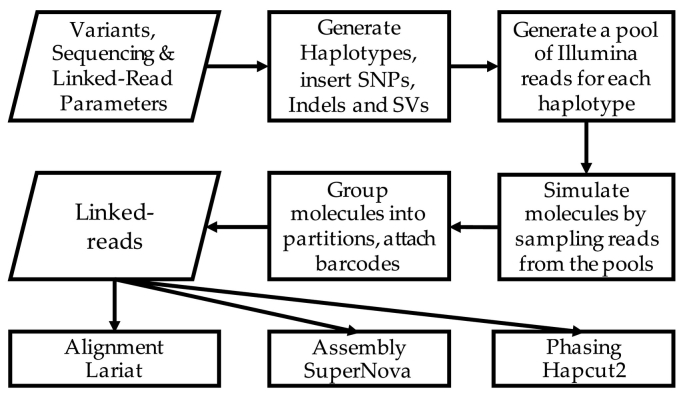
LRSim workflow. Lariat, SuperNova and HapCUT2 are three tools downstream to LRSim. Lariat is an aligner module of LongRanger specified for linked-read alignment. SuperNova is a genome assembler specified for lined-read. HapCUT2 is a phasing algorithm that works with linked-read. LRSim provides an option to skip variant simulation with SURVIVOR and take a user-provided variant file.

Fig. 8 shows the overview of the LRSim workflow. Briefly, LRSim first uses SURVIVOR [14] to simulate homozygous and heterozygous SNPs, indels and structural variants within the user-specified genome sequence. Second, LRSim uses DWGSIM (GitHub: nh13/DWGSIM) to mimic the error profile of Illumina paired-end reads and generates 50% more reads than the user requested. Default parameters for running DWGSIM are shown in the Additional file 1: Supplementary Note. PCR duplicates are not simulated. Third, considering the Illumina reads as a pool, LRSim emulates the process of linked-read sequencing by attaching barcodes to reads randomly selected from the pool. It is worth mentioning that LRSim allows both user-specified genome variants, or rely on SURVIVOR to randomly simulate SNPs, indels and structural variants at a user specified rate. The DWGSIM we used in LRSim downstream to SURVIVOR is also capable in simulating SNPs and indels, but not structural variants. Users can also disable SURVIVOR and enable DWGSIM for variant simulation by change just a few lines of code.

LRSim is available open-source in Github under an MIT License. The LRSim pipeline is written in perl, and uses a few genomics tools as components for extracting sequences and simulating reads including SAMtools, SURVIVIOR, and DWGSIM. For a human genome using default parameters, the memory consumption peaks at 48 GB, and starting from scratch takes about five hours to finish using 8 threads, or 1.5 hours if only the linked-reads related parameters *x*, *f*, *t* or *m* are altered, thus avoiding rerunning the expensive DWGSIM stage. In exchange for lower memory consumption, longer running time is required. The peak memory can be further decreased by decreasing the copies of DWGSIM run in parallel.

## Declarations

### Acknowledgements

We thank Steven Salzberg for his valuable comments and editing the manuscript. We also thank the team of 2016 CSHL-NCBI Hackathon for helpful discussions. We would also like to thank Deanna Church, David Jaffe, and Patrick Marks from 10X Genomics for their helpful discussions during the development of LRSim. We would also like to thank the MaizeCode Project for providing access to the maize sequencing data.

### Funding

This work has been supported by the NSF [DBI-1350041 and IOS-1445025] and the NIH [R01-HG006677] to Michael C. Schatz.

### Availability of Data and Materials

Project name: LRSim.

Project homepage: https://github.com/aquaskyline/LRSIM

Archived version: https://github.com/aquaskyline/LRSIM/releases/tag/1.0

Example scripts: https://github.com/aquaskyline/LRSIM/tree/master/test

Operating system: Platform independent.

Programming language: Perl and C ++.

Other requirements: See GitHub page.

License: MIT.

DOI: https://doi.org/10.5281/zenodo.808913, 10.24433/CO.8e25703d-92df-4eb5-8683-d1108100b39c.

Any restrictions to use by non-academics: None.

### Authors' Contribution

RL, FJS and MCS conceived the study. RL developed and implemented the LRSim algorithm. RL, SMK and CAD performed the evaluation on different parameters. CAD modified the SURVIVOR software package to allow for SNP simulation. RL, FJS and MCS wrote the paper. All authors have read and approved the final version of the manuscript.

### Competing Interests

The authors declare that they have no competing interests.

## Appendix A Supplementary Data

Supplementary material Image 2
