# Supplementary material for: LRSim: A Linked-Reads Simulator Generating Insights for Better Genome Partitioning
Source: Comput Struct Biotechnol J. 2017 Nov 9;15:478–84. doi: 10.1016/j.csbj.2017.10.002 (PMC5711661; doi:10.1016/j.csbj.2017.10.002)
Supplement: Supplementary file 1 — Supplementary material [file mmc1.docx]

LRSim: a Linked Reads Simulator generating insights for better genome partitioning

Ruibang Luo, Fritz J. Sedlazeck, Charlotte A. Darby, Stephen M. Kelly, Michael C. Schatz

**Supplementary Table 1**, a) Barcode mismatch profile collected from 9 genomes (Chinese Trios HG00512/3/4, Puerto Rican Trio HG00731/2/3 and Yoruba Trio NA19238/39/40). Mismatches were detected as the difference between the BX and RX tag for each read available in the BAM file produced by 10x Genomics’ analysis pipeline for Chromium sequencing output called ‘LongRanger’. The BX tag stands for ‘Chromium barcode sequence that is error-corrected and confirmed against a list of known-good barcode sequences’. RX stands for ‘Raw Chromium barcode sequence. This read is subject to sequencing errors’; b) barcode error rate suggested by base quality in the 9 genomes.

a.

b.

**Supplementary Table 2**, a) the count of top 100 mostly reads supported barcodes in the 13 samples; b) the top 100 mostly supported barcodes in each sample and the count of supporting reads. This is an informative table but not described in the maintext.

a.

b.

**Supplementary Table 3**, the total length of low coverage regions of a) Chromium 80x NA12878 dataset; and 2) Illumina 300x NA12878 dataset available in Supplementary Note 1.

a.

b.

**Supplementary Figure 1**, a. Sequencing coverage per chromosome of the 13 samples; b. The average coverage per 1Mbp bin of chromosome 21 of the 13 samples. The coverages were normalized to the sample with the lowest average coverage (NA24149, 30.36x). Further investigation by realigning the reads from the excessive high depth region at chr21:8,219,888-8,466,847 again to the whole genome as single-end reads shows that about 50% of the reads can be aligned to the alternative contigs and other chromosomes.

a.

b.

**Supplementary Figure 2**, GC bias of the 13 samples.

**Supplementary Note**

Generating molecule details per partition

1. For each barcode, generate a bam file with reads with the BX tag equals to the barcode.
2. For each bam file ${barcode}, do

samtools view -u ${barcode}.bam 2>/dev/null| samtools sort -o - - 2>/dev/null | samtools targetcut -Q 1 -0 '-1' -2 24 - 2>/dev/null > ${barcode}.molecules.sam

1. For each .molecules.sam file, count the number of rows as the number of molecules per partition, count the length of column 10 as the length of each molecule.

Default parameters for running DWGSIM

dwgsim -N ${numReadsPerHaplotype} -e 0.0001,0.0016 -E 0.0001,0.0016 -d 350 -s 35 -1 135 -2 151 -H -y 0 -S 0 -c 0 -m /dev/null ${inputFasta} ${outputPrefix}

Links to datasets

NIST NA12878 10x data:

<ftp://ftp-trace.ncbi.nlm.nih.gov/giab/ftp/data/NA12878/10Xgenomics_ChromiumGenome/NA12878_GRCh38.bam>

Ashkenazim Trio NA24385/149/143:

<ftp://ftp-trace.ncbi.nlm.nih.gov/giab/ftp/data/AshkenazimTrio/analysis/10XGenomics_ChromiumGenome_LongRanger2.0_06202016/HG002_NA24385_son/NA24385_GRCh38.bam>

<ftp://ftp-trace.ncbi.nlm.nih.gov/giab/ftp/data/AshkenazimTrio/analysis/10XGenomics_ChromiumGenome_LongRanger2.0_06202016/HG003_NA24149_father/NA24149_GRCh38.bam>

<ftp://ftp-trace.ncbi.nlm.nih.gov/giab/ftp/data/AshkenazimTrio/analysis/10XGenomics_ChromiumGenome_LongRanger2.0_06202016/HG004_NA24143_mother/NA24143_GRCh38.bam>

Chinese Trios HG00512/3/4:

<http://ftp.1000genomes.ebi.ac.uk/vol1/ftp/data_collections/hgsv_sv_discovery/working/20160915_10x_genomics_svs/HG00512_phased_possorted_bam.bam>

<http://ftp.1000genomes.ebi.ac.uk/vol1/ftp/data_collections/hgsv_sv_discovery/working/20160915_10x_genomics_svs/HG00513_phased_possorted_bam.bam>

<http://ftp.1000genomes.ebi.ac.uk/vol1/ftp/data_collections/hgsv_sv_discovery/working/20160915_10x_genomics_svs/HG00514_phased_possorted_bam.bam>

Puerto Rican Trio HG00731/2/3:

<http://ftp.1000genomes.ebi.ac.uk/vol1/ftp/data_collections/hgsv_sv_discovery/working/20160915_10x_genomics_svs/HG00731_phased_possorted_bam.bam>

<http://ftp.1000genomes.ebi.ac.uk/vol1/ftp/data_collections/hgsv_sv_discovery/working/20160915_10x_genomics_svs/HG00732_phased_possorted_bam.bam>

<http://ftp.1000genomes.ebi.ac.uk/vol1/ftp/data_collections/hgsv_sv_discovery/working/20160915_10x_genomics_svs/HG00733_phased_possorted_bam.bam>

Yoruba Trio NA19238/39/40:

<http://ftp.1000genomes.ebi.ac.uk/vol1/ftp/data_collections/hgsv_sv_discovery/working/20160915_10x_genomics_svs/NA19238_phased_possorted_bam.bam>

<http://ftp.1000genomes.ebi.ac.uk/vol1/ftp/data_collections/hgsv_sv_discovery/working/20160915_10x_genomics_svs/NA19239_phased_possorted_bam.bam>

<http://ftp.1000genomes.ebi.ac.uk/vol1/ftp/data_collections/hgsv_sv_discovery/working/20160915_10x_genomics_svs/NA19240_phased_possorted_bam.bam>

Illumina 300x NA12878

<ftp://ftp-trace.ncbi.nlm.nih.gov/giab/ftp/data/NA12878/NIST_NA12878_HG001_HiSeq_300x/NHGRI_Illumina300X_novoalign_bams/HG001.hs37d5.300x.bam>

Two NA12878 raw read datasets generated by 10x Genomics

<https://support.10xgenomics.com/de-novo-assembly/datasets/msNA12878>

Phased variants of NA12878

https://support.10xgenomics.com/genome-exome/datasets/NA12878_WGS_210
